# Downregulation of m^6^A writer complex member METTL14 in bladder urothelial carcinoma suppresses tumor aggressiveness

**DOI:** 10.1002/1878-0261.13181

**Published:** 2022-03-24

**Authors:** Catarina Guimarães‐Teixeira, João Lobo, Vera Miranda‐Gonçalves, Daniela Barros‐Silva, Cláudia Martins‐Lima, Sara Monteiro‐Reis, José Pedro Sequeira, Isa Carneiro, Margareta P. Correia, Rui Henrique, Carmen Jerónimo

**Affiliations:** ^1^ Cancer Biology and Epigenetics Group Research Center of IPO Porto (CI‐IPOP)/RISE@CI‐IPOP (Health Research Network) Portuguese Oncology Institute of Porto (IPO Porto)/Porto Comprehensive Cancer Center (Porto.CCC) Portugal; ^2^ PhD Programme in Pathology & Molecular Genetics School of Medicine & Biomedical Sciences–University of Porto (ICBAS‐UP) Portugal; ^3^ Department of Pathology Portuguese Oncology Institute of Porto (IPOP) Portugal; ^4^ Department of Pathology and Molecular Immunology School of Medicine & Biomedical Sciences–University of Porto (ICBAS‐UP) Portugal

**Keywords:** bladder cancer, epitranscriptome, m6A‐regulators proteins, METTL14, N6‐methyladenosine, RNA modifications

## Abstract

N6‐methyladenosine (m^6^A) and its regulatory proteins have been associated with tumorigenesis in several cancer types. However, knowledge on the mechanistic network related to m^6^A in bladder cancer (BlCa) is rather limited, requiring further investigation of its functional role. We aimed to uncover the biological role of m^6^A and related proteins in BlCa and understand how this influences tumor aggressiveness. N6‐adenosine‐methyltransferase catalytic subunit (METTL3), N6‐adenosine‐methyltransferase noncatalytic subunit (METTL14), protein virilizer homolog (VIRMA), and RNA demethylase ALKBH5 (ALKBH5) had significantly lower expression levels in BlCa compared to that in normal urothelium. *METTL14* knockdown led to disruption of the remaining methyltransferase complex and a decrease in m^6^A abundance, as well as overall reduced tumor aggressiveness (decreased cell invasion and migration capacity and increased apoptosis). Furthermore, *in vivo*, *METTL14* knockdown caused tumor size reduction. Collectively, we propose methyltransferase METTL14 as a key component for m^6^A RNA deposit and that it is closely related to BlCa progression, playing an important role in tumor aggressiveness. These data contribute to a better understanding of the m^6^A writer complex, which might constitute an appealing therapeutic target.

## Graphical abstract

We identified several N6‐methyladenosine (m^6^A)‐regulator proteins dysregulated in bladder cancer (BlCa). *METTL14* knockdown disrupted the methyltransferase complex and decreased m^6^A abundance, impairing tumor aggressiveness. Furthermore, chorioallantoic membrane assay revealed significant tumor size reduction after knockdown of *METTL14 in vivo*. Our results suggest that METTL14 is a key component for m^6^A RNA deposit and is implicated in BlCa progression.

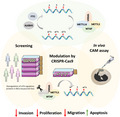

**Abbreviations**
ALKBH5: alkB homologue 5
BlCa: bladder cancer
BUC: bladder urothelial carcinoma
FITC: fluorescein isothiocyanate
FTO: human obesity‐associated
IgG: immunoglobulin G
IHC: immunohistochemistry
m^6^A: N6‐methyladenosine
METTL14: methyltransferase‐like protein 14
METTL3: methyltransferase‐like protein 3
MIBC: muscle invasive bladder cancer
NMIBC: nonmuscle invasive bladder cancer
NUT: normal urothelial tract
RBM15/15B: RNA‐binding motif proteins 15/15B
TBS: tris‐buffered saline
TCGA: The Cancer Genome Atlas
UCs: urothelial carcinomas
VIRMA: virilizer
WB: Western blot
WTAP: Wilm's tumor‐associated protein
ZC3H13: zinc finger CCCH domain‐containing

## 1 Introduction

Bladder cancer (BlCa) is the tenth most incident cancer worldwide and the fourteenth leading cause of death from cancer, according to GLOBOCAN 2020 [1]. Most BlCa cases (> 90%) arise from the inner lining of the urinary tract (urothelium) and are commonly designated urothelial carcinomas (UCs). BlCa is further classified into two main categories, with distinct pathobiology and clinical implications: nonmuscle‐invasive BlCa (NMIBC) and muscle‐invasive BlCa (MIBC). NMIBC accounts for approximately 75% of all diagnosed BlCa cases. Although these tumors usually do not represent an immediate survival threat, they often recur. Moreover, 10–30% of patients with NMIBC eventually progress to MIBC. MIBC represents about 25% of BlCa cases, is more clinically aggressive and may rapidly progress and metastasize [2, 3].

At the molecular level, several classifications of BlCa (MIBC, specifically) have been proposed, and all of them are partially coincident, allowing to discriminate among two major molecular subtypes: luminal and basal‐like BlCa [4, 5]. More recently, these subtypes have been stratified into five additional groups, with putative prognostic and therapeutic implications [6]. Nonetheless, BlCa remains a deadly disease, and clinical care of these patients represents a major economic burden [7]. Therefore, there is an urgent need to understand the mechanisms of BlCa progression, allowing for development of new diagnostic, monitoring, and therapeutic strategies.

Over the last decade, the rapid improvement of a vast array of techniques entailed the flourishment of genomic and epigenomic studies, increasing our knowledge about tumor‐specific alterations. More recently, a new layer of gene expression regulation at RNA level was identified. Epitranscriptomics refers to the study of reversible chemical modifications affecting RNA molecules, including both mRNA and noncoding RNAs [8, 9, 10]. N6‐methyladenosine (m^6^A, i.e., the methylation of the adenosine base at the nitrogen‐6 position) is the most abundant internal chemical modification of mRNAs in eukaryotes. m^6^A is specifically enriched near the stop codon, at 3′‐UTR, and within long internal exons, thereby affecting different steps of mRNA's lifespan, such as transcription, splicing, nuclear export, and translation [11]. This modification is catalyzed by the m^6^A methyltransferase complex‐MTC (‘writers’), which is composed by methyltransferase‐like 3 and 14 proteins (METTL3 and METTL14) and their cofactors: Wilms tumor‐associated protein (WTAP), Virilizer (KIAA1429/VIRMA), RNA‐binding motif proteins 15/15B (RBM15/15B), and zinc finger CCCH domain‐containing protein 13 (HAKAI and ZC3H13) [12, 13]. METTL3 and METTL14 constitute a heterodimer that synergistically introduces m^6^A. Although METTL3 has been reported to play a central role in MTC stability, data on METTL14 function is notably lacking [12, 14]. m^6^A can also be removed, by means of m^6^A demethylases (‘erasers’), including the human obesity‐associated protein (FTO) and AlkB homologue 5 (ALKBH5) [15, 16]. Additionally, there are proteins that bind directly to the m^6^A mediating its function, known as ‘readers’, including the YTH domain family and HNRNPA2B1 or IGF2BP family of proteins [17, 18, 19].

Remarkably, several studies have implicated m^6^A and associated regulatory proteins in several cancers, including those of the prostate, breast, lung, brain, liver, colorectal, testis, and endometrium, as well as leukemia [20, 21, 22, 23, 24, 25, 26]. Nonetheless, knowledge of the mechanistic network underlying m^6^A (de)regulation in BlCa is still limited. Moreover, further investigation on the role of this modification in BlCa may uncover novel prognostic and/or predictive biomarkers that might be useful for patient management [27]. Thus, we sought to assess the expression patterns of m^6^A and its associated proteins in BlCa primary tumors and cell lines, as well as the specific role of METTL14 in BlCa tumorigenesis and aggressiveness.

## 2 Materials and methods

### 2.1 *In silico* analysis

To evaluate the differential expression of m^6^A writers and erasers in BlCa tissues, the online platform cBio‐Portal (https://www.cbioportal.org/); last access on 12/01/2019) was accessed [28] with the user‐defined entry gene set ‘*METTL3, METTL14, VIRMA, WTAP, ALKBH5 and FTO*’. The Cancer Genome Atlas (http://cancergenome.nih.gov) database was selected to determine which subunit might play the crucial role of m^6^A deregulation in BlCa.

Gene Expression Profiling Interactive Analysis (GEPIA) web server (http://gepia.cancer‐pku.cn/); last access on 12/01/2019) was used for analyzing the RNA sequencing expression data of bladder tumors and normal samples from the TCGA and GTEx projects using a standard processing pipeline: |log2FC| Cut‐off = 1; *P*‐value cutoff = 0.01 [29].

### 2.2 Patients and sample collection

One hundred and twenty (60 NMIBCs and 60 MIBCs) formalin‐fixed and paraffin‐embedded tissues were collected from the archives of the Department of Pathology of Portuguese Oncology Institute of Porto. These samples are representative of primary bladder UC, without any prior treatment and were diagnosed between 1997 and 2005. Additionally, 40 tissue samples of normal urothelial tract mucosa (NUT), originating from nephrectomy specimens of patients with renal cell tumors (either renal cell carcinoma or oncocytoma) and no history of UC, were selected.

Clinical files and pathology reports were reviewed. All histological slides (of primary tumors) were reviewed by a dedicated pathologist and tumors were reclassified in light of the most recent 2016 World Health Organization (WHO) Classification of Tumors of the Urinary System and Male Genital Organs [30]. Staging was performed according to the 8th edition of the American Joint Committee on Cancer (AJCC) staging manual [31].

This study was approved by the Ethics Committee (CES‐IPO 372/2017) of Portuguese Oncology Institute of Porto, Portugal. Informed consent was obtained from all patients. All procedures performed in tasks involving human participants were in accordance with the ethical standards of the institutional and/or national research committee and with the 1964 Helsinki Declaration and its later amendments or comparable ethical standards.

### 2.3 Immunohistochemistry

Immunohistochemistry (IHC) analysis for m^6^A modification, m^6^A writers METTL3, METTL14, VIRMA, and WTAP, erasers ALKBH5 and FTO was performed using the Novolink™ Max Polymer Detection System (Leica Biosystems, Wetzlar, Germany). The procedure was performed as previously described by our research team [23]. Appropriate positive controls were used for each antibody (Table S1).

A semiquantitative analysis of immunoexpression was performed by an experienced pathologist and categorized according to intensity and percentage of stained cells in the slide (between 0% and 100%). The following scores were used for further analysis: intensity score, consisting of score 0 (absent immunoexpression), score 1+ (immunoexpression only barely discernible at high power magnification), score 2+ (immunoexpression well discernible at high power but faint in low power magnification), and score 3+ (strong immunoexpression well discernible at low power magnification); and percentage score, consisting of score 0 (< 1% of immunoreactive cells), score 1+ (< 50% of immunoreactive cells), score 2+ (50–75% of immunoreactive cells), and score 3+ (75–100% of immunoreactive cells). The final staining score was calculated by multiplying intensity and percentage score, resulting in a combined score value ranging from 0 to 9+.

### 2.4 Bladder cancer cell lines and cell culture

Seven BlCa cell lines (MGHU3, RT112, 5637, J82, T24, UMUC3, TCCSUP) and one additional normal bladder cell line SV‐HUC1 from American Type Culture Collection (ATCC, Manassas, VA, USA) were used for m^6^A‐regulators' proteins characterization (Table S2).

All culture media were supplemented with 10% FBS (Biochrom, MERK, Berlin, Germany) and 1% penicillin/streptomycin (GIBCO^®^, Invitrogen, Waltham, MA, USA). Cells were maintained at 37 °C with 5% CO_2_ and routinely tested for *Mycoplasma* sp. contamination using a PCR‐based universal mycoplasma detection kit (PCR Mycoplasm Detection Set; Clontech Laboratories, Oxford, UK).

### 2.5 Protein extraction and quantification

Total protein was extracted from cells using radioimmuno precipitation assay buffer (Santa Cruz Biotechnology Inc., Dallas, TX, USA) with 10% of protein inhibitor cocktail. After 15 min on ice, samples were centrifuged at 18928 g during 30 min at 4 °C and the supernatant was collected. Then, protein was quantified using a Pierce BCA Protein Assay Kit (Thermo Scientific Inc., Waltham, MA, USA), according to the manufacturer's instructions.

### 2.6 Western blot

Western blot (WB) analysis was performed as previously reported [32]. Primary antibodies used and respective dilutions are depicted in Table S3. Quantification was performed using band densitometry analysis from the imagej software (version 1.6.1; National Institutes of Health, Bethesda, MD, USA), by comparing the specific protein band intensity with the loading control beta‐actin (β‐ACT).

### 2.7 Immunofluorescence analysis

Cells were seeded in coverslips in 24‐well plates at 25 000 cells/well (previously optimized concentration) and allowed to adhere at 37 °C, 5% CO_2_ overnight. On the next day, cells were fixed 4% paraformaldehyde for 10 min and permeabilized with 0.25% Triton X‐100 solution in PBS for 15 min. After that, cells were blocked with 5% BSA for 30 min, followed by primary antibody incubation at specific dilution (Table S3), overnight at RT.

Following primary antibody, cells were incubated with secondary antibody anti‐rabbit immunoglobulin G (IgG; Alexa Fluor™ 488 goat, A11008; Invitrogen) or anti‐mouse IgG‐fluorescein isothiocyanate (FITC goat SLB4878; Sigma‐Aldrich™, St. Louis, MO, USA) for 1 h, at RT (Table S3). Nuclear staining was performed with 4′,6‐diamidino‐2‐phenylindole (DAPI) (AR1176; BOSTER Biological Technologies, Pleasanton, CA, USA) in mounting medium.

### 2.8 RNA methylation quantification

RNA was extracted from cell lines by TripleXtractor (GRiSP^®^, Porto, Portugal) according to the manufacturer's recommended protocol. To detect m^6^A levels, m^6^A RNA Methylation Quantification Kit (ab185912; Abcam, Cambridge, UK) was used as indicated by the manufacturers. In this assay, the m^6^A is detected using capture and detection antibodies. The detected signal is enhanced and then quantified using colorimetric methodology by reading the absorbance in a microplate spectrophotometer (Fluostar Omega; BMG Labtech, Ortenberg, Germany). The amount of m^6^A is proportional to the optical density (OD) intensity measured.

### 2.9 CRISPR‐Cas9‐mediated knockdown of METTL14

After protein analysis by WB, UMUC3 cell line was chosen to perform METTL14 gene knockdown by plasmids carrying the CRISPR‐Cas9 system containing a guide RNA sequence targeting METTL14 (GenScript, Piscataway, NJ, USA). For plasmid transfection, Lipofectamine^®^ 3000 reagent (Invitrogen) was used according to the manufacturers' instructions. Cells were transfected with the plasmid for 8 h, followed by selection of cells which incorporated the CRISPR‐Cas9 system with 1 μg of puromycin for each 1mL of cell culture medium. As experimental controls, we included WT cells with lipofectamine to control for the effect of the transfection reagent and cells transfected with a scrambled (nonspecific guide) vector, used as controls for all experiments (to control for off‐target effects).

After selection, cells were expanded, and total protein was extracted to confirm METTL14 protein downregulation (METTL14‐KD). UMUC3 will type cells were used as controls for WB analysis.

### 2.10 Dot blot

For m^6^A dot blot, 2 µg of previously extracted RNA from METTL14‐KD and scramble cells were spotted onto nitrocelulose membrane. The samples were crosslink with UV light, followed by m^6^A antibody incubation (1 : 1000 dilution in TBS‐T, supplemented with 5% milk) and subsequent HRP‐conjugated secondary antibody (1 : 5000 dilution in TBS‐T, supplemented with 5% milk). Finally, the samples were detected with 3,3′‐diaminobenzidine. For loading control, 0.02% methylene blue (MB) was used to stain the same RNA samples.

### 2.11 Cell viability assay

For the viability assay, resazurin (Canvax Biotech, Córdoba, Spain) was used, according to the manufacturer's instructions and a previous report by our group [33]. Cells were plated into 96‐well plates in complete DMEM at density of 6000 cells/well and incubated overnight, at 37 °C in 5% CO_2_. Then, cells were maintained from 0 h until 72 h. Results were normalized to the scramble condition. All experiments were performed in biological triplicates, each with experimental triplicates.

### 2.12 Apoptosis assay

To access the apoptotic effect of METTL14‐KD on UMUC3 cell lines, the FITC Annexin V Apoptosis Detection Kit with 7‐aminoactinomycin D (7AAD) (Biolegend, San Diego, CA, USA) was used according to the manufacturer's instructions. For that, 250  000 cells were used, harvested for cell staining, and acquired in FACS Canto™ II Cell Analyzer (BD Biosciences, Franklin Lakes, NJ, USA). Afterward, the data were analyzed using the flowjo™ software (Ashland, OR, USA) and the values were compared to scramble UMUC3.

### 2.13 Wound healing assay

For migration capacities, cells were seeded (6‐well plate) in complete DMEM at an optimal density to obtain at least 95% of confluence in the next 24 h and incubated at 37 °C, 5% CO_2_. The procedure and the calculation formula were performed as previously described [34].

The wound areas were photographed in two specific sites at 40× magnification using an Olympus IX51 inverted microscope equipped with an Olympus XM10 Digital Camera System (Olympus, Tokyo, Japan). At least three independent experiments were performed.

### 2.14 Invasion assay

Cell invasion was evaluated by using 24‐well BD BioCoat Matrigel Invasion Chambers, with 8 μm pore size membranes (BD BioSciences, Franklin Lakes, NJ, USA). Thirty thousand cells/insert were seeded, and the procedure was done as published elsewhere [35].

### 2.15 Proximity ligation assay

METTL14‐KD UMUC3 cells were cultured in 1 cm^2^ coverslips, and scramble was used as controls. Cells were fixed in 4% formaldehyde (Sigma, St. Louis, MO, USA) for 10 min and permeabilized in 0.5% Triton X‐100 (Sigma, St. Louis, MO, USA), for 5 min, at room temperature and gently stirred.

Proximity ligation assay was performed using the commercial kit Duolink *In Situ* (OLINK Bioscience, Uppsala, Sweden), according to the manufacturer's instructions. The antibodies used were METTL14 (ab220031) and METTL3 (ab195352). After the procedure, slides were evaluated under a fluorescence microscope (Olympus IX51; Olympus, Tokyo, Japan).

### 2.16 Chorioallantoic membrane assay

Twenty‐two fertilized chicken eggs (Pinto Bar, Amares, Braga, Portugal) were incubated at 37 °C in a humid environment. On the third day of development, a window was opened into the eggshell under aseptic conditions. On day 10, UMUC3 scramble and UMUC3 METTL14‐KD cell suspensions in growth factor‐reduced Matrigel (BD Biosciences) were seeded on the chorioallantoic membrane (CAM). Then, on day 17, the chicken embryos were sacrificed by eggs' incubation at −80 °C for 10 min and tumors were dissected formalin‐fixed and included in paraffin. Relative size *in ovo* condition was assessed using cellsens software (version V0116; Olympus). *Ex ovo* pictures were also obtained for blood vessels counting using imagej software.

### 2.17 Statistical analysis

Statistical analysis was performed using the graphpad prim 7.0 software (GraphPad Software Inc., Chicago, IL, USA) and ibm ^®^ spss ^®^ statistic software version 23 (IBM‐SPSS Inc., La Jolla, CA, USA). Nonparametric Mann–Whitney *U*‐test or Kruskal–Wallis tests were used to compare the distribution of continuous variables among groups, as appropriate. Bonferroni's correction was employed in case of multiple testing. Differences in immunoexpression of the several targets between NUT and BUC tissues were assessed by chi‐square or Fisher's exact test. Correlation between continuous variables was assessed with Spearman's (*r*_S_) nonparametric correlation test.

*P*‐values were considered statistically significant when inferior to 0.05. Significance is shown vs. the respective control and depicted as follows: *P ≤ 0.05, **P < 0.01, ***P < 0.001, ****P < 0.0001, and *P* > 0.05 (ns—nonsignificant).

## 3 Results

### 3.1 *In silico* analysis of m^6^A methylases and demethylases in bladder cancer

**Fig. 1:**
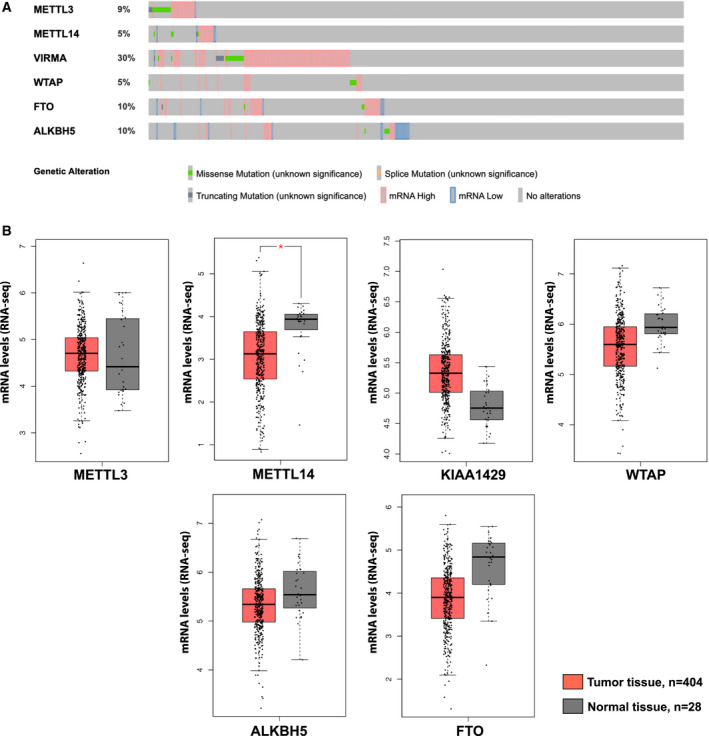
*In silico* analysis. (A) frequency of alterations in queried genes of TCGA database; (B) mRNA expression levels of m6A modifying enzymes in clinical tissue samples of *The Cancer Genome Atlas* (TCGA) and GTEx bladder (*n* = 404, red boxplot) and adjacent normal specimens (*n* = 28, gray boxplot). Notice the METTL14 mRNA expression was the only m6A‐regulators' proteins significantly downregulated in BlCa to other queried genes (*P < 0.05). ANOVA test was used, genes were considered differentially expressed when expression levels were changed at least twofold (log2 fold‐change threshold value equal to or bigger than 1) and had an Benjamini and Hochberg false discovery rate adjusted *q*‐value smaller then 0.01.

*The Cancer Genome Atlas* (TCGA) database available for analysis at cBioPortal [28] includes tumor samples from 413 patients with MIBC. Seventy‐four percent were males, whereas 26% were females, with a median age at diagnosis of 61 years. *In silico* analysis of the genomic regions encoding m^6^A‐regulators proteins displayed different molecular alterations in 56% of MIBC patients (Fig. 1A). From all the queried m^6^A‐regulators proteins, VIRMA was the most frequently altered gene (30% of the samples) in these tumors, being frequently amplified or expressing high mRNA levels. Next, we examined the expression levels in the same set of samples comparing with noncancerous tissues, using the *Gene Expression Profiling Interactive Analysis* (GEPIA) server [29]. METTL14 mRNA expression was the only m^6^A‐regulators proteins from the analyzed panel significantly downregulated in BlCa compared to the normal bladder tissues (Fig. 1B).

### 3.2 Immunoexpression profiles of m^6^A and m^6^A‐regulators proteins in BlCa tissues

Since, no data are available for NMIBC and validation is lacking for MIBC, m^6^A‐machinery components were further assessed in a different and independent tissue set from bladder cancer patients diagnosed and treated at Portuguese Oncology Institute of Porto (IPO Porto) (Table 1).

**Table 1:** Clinicopathological parameters of the bladder cancer patients. BUC, bladder urothelial carcinomas; n.a., not applicable; NUT, normal urothelial tract.

Clinicopathological features	BUC	NUT
Patients, *n*	120	40
Gender, *n* (%)		
Male	93 (77.5%)	25 (62.5%)
Female	27 (22.5%)	15 (37.5%)
Median age, years (range)	69 (43–89)	63 (40–87)
MIBC and NMIBC, *n* (%)		
Muscle invasive	60 (50%)	n.a.
Nonmuscle invasive	60 (50%)	n.a.
Pathological stage, *n* (%)		
pTa	20 (16.7%)	n.a.
pT1	40 (33.3%)	n.a.
pT2	27 (22.5%)	n.a.
pT3	21 (17.5%)	n.a.
pT4	11 (9.2%)	n.a.
pTx	1 (0.8%)	n.a.

**Fig. 2:**
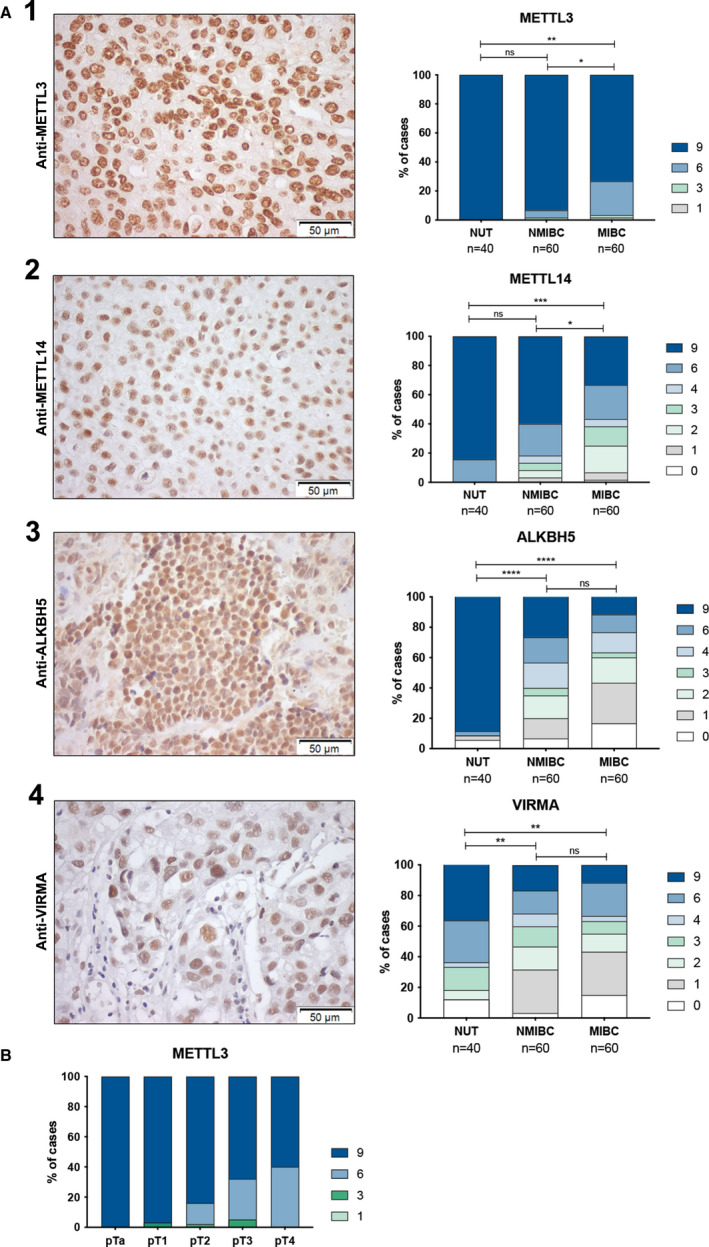
IHC in tissue samples. (A) Illustrative images of immunostaining and comparison of METTL3, METLL14, VIRMA, and ALKBH5 in normal and bladder cancer tissues: (1) METTL3 nuclear immunoexpression in bladder cancer (NMIBC vs. MIBC *P* = 0.023; NUT vs. NMIBC *P* = 0.005); (2) METTL14 nuclear immunoexpression in bladder cancer (NMIBC vs. MIBC *P* = 0.049; NUT vs. MIBC *P* = 0.003); (3) VIRMA nuclear immunoexpression in bladder cancer (NUT vs. NMIBC *P* = 0.003; NUT vs. MIBC *P* = 0.006); (4) ALKBH5 nuclear immunostaining in bladder cancer (NUT vs. NMIBC *P* < 0.0001; NUT vs. MIBC *P* < 0.0001). (B) Association between METTL3 protein level and pathological stage. Immunostaining based on h‐score (ranges from 0, +1, +2, +3, +4, +6, +9); *n* = 60 NMIBCs; *n* = 60 MIBCs; *n* = 40 NUT. Qui‐square, *P < 0.05, **P < 0.01, ***P < 0.001, ****P < 0.0001; ns, not significant. NMIBC, nonmuscle invasive bladder cancer; MIBC, muscle invasive bladder cancer; NUT, normal urothelial tract. All pictures were taken at 200× magnification, 50 µm.

Nuclear expression was observed for all tested proteins. Illustrative examples of immunostaining patterns are depicted in Fig. 2A and Fig. S1. Overall, METTL3, METTL14, VIRMA, and ALKBH5 immunoexpression levels differed significantly between tumor and normal tissues. Specifically, METTL3 (*P* = 0.0030), METTL14 *(P* = 0.0019), ALKBH5 (*P* < 0.0001), and VIRMA (*P* = 0.0081) (Fig. 2A) disclosed significantly lower expression in BlCa compared to normal tissues. Moreover, METTL3 and METTL14 (heterodimeric catalytic core) showed significantly lower expression levels in MIBC comparing with NMIBC (*P* = 0.0227 and *P* = 0.0489, respectively). Nonetheless, no significant differences were apparent regarding m^6^A, WTAP and FTO immunostaining among the three tissue sample groups.

The expression of all studied writers positively correlated with m^6^A abundance in tumor samples. The same was observed regarding erasers expression, except for FTO. Interestingly, correlations were also observed between most of the m^6^A writers and erasers expression, except for WTAP and both erasers, as well as between VIRMA and FTO (Table 2)

**Table 2:** Correlation between different regulatory proteins. Spearman's rank correlation coefficient (rho).

	m^6^A	METTL3	METTL14	VIRMA	WTAP	FTO	ALKBH5
m^6^A	—	***P* = 0.001** ***R* = 0.488**	***P* < 0.0001** ***R* = 0.306**	***P* = 0.015** ***R* = 0.223**	***P* = 0.035** ***R* = 0.194**	*P* = 0.081 *R* = 0.162	***P* = 0.014** ***R* = 0.225**
METTL3	—	—	***P* < 0.0001** ***R* = 0.435**	***P* < 0.0001** ***R* = 0.343**	***P* < 0.0001** ***R* = 0.415**	***P* = 0.014** ***R* = 0.014**	***P* = 0.001** ***R* = 0.294**
METTL14	—	—	—	***P* < 0.0001** ***R* = 0.373**	***P* = 0.008** ***R* = 0.243**	*P* = 0.066 *R* = 0.169	***P* < 0.0001** ***R* = 0.458**
VIRMA	—	—	—	—	***P* = 0.001** ***R* = 0.289**	*P* = 0.309 *R* = 0.094	***P* < 0.0001** ***R* = 0.416**
WTAP	—	—	—	—	—	*P* = 0.342 *R* = 0.089	*P* = 0.131 *R* = 0.140
FTO	—	—	—	—	—	—	*P* = 0.869 *R* = −0.015
ALKBH5	—	—	—	—	—	—	—

Bold values represent statistically significant correlations.

No statistically significant differences in expression were found for any of the tested proteins and patient smoking habits, gender, or age at diagnosis. Nonetheless, a significant association was found between METTL3 expression and pathological stage. Indeed, decreased METTL3 expression was apparent in advanced disease (Fig. 2B).

### 3.3 Cellular localization and quantification of m^6^A and regulatory proteins in cell lines

**Fig. 3:**
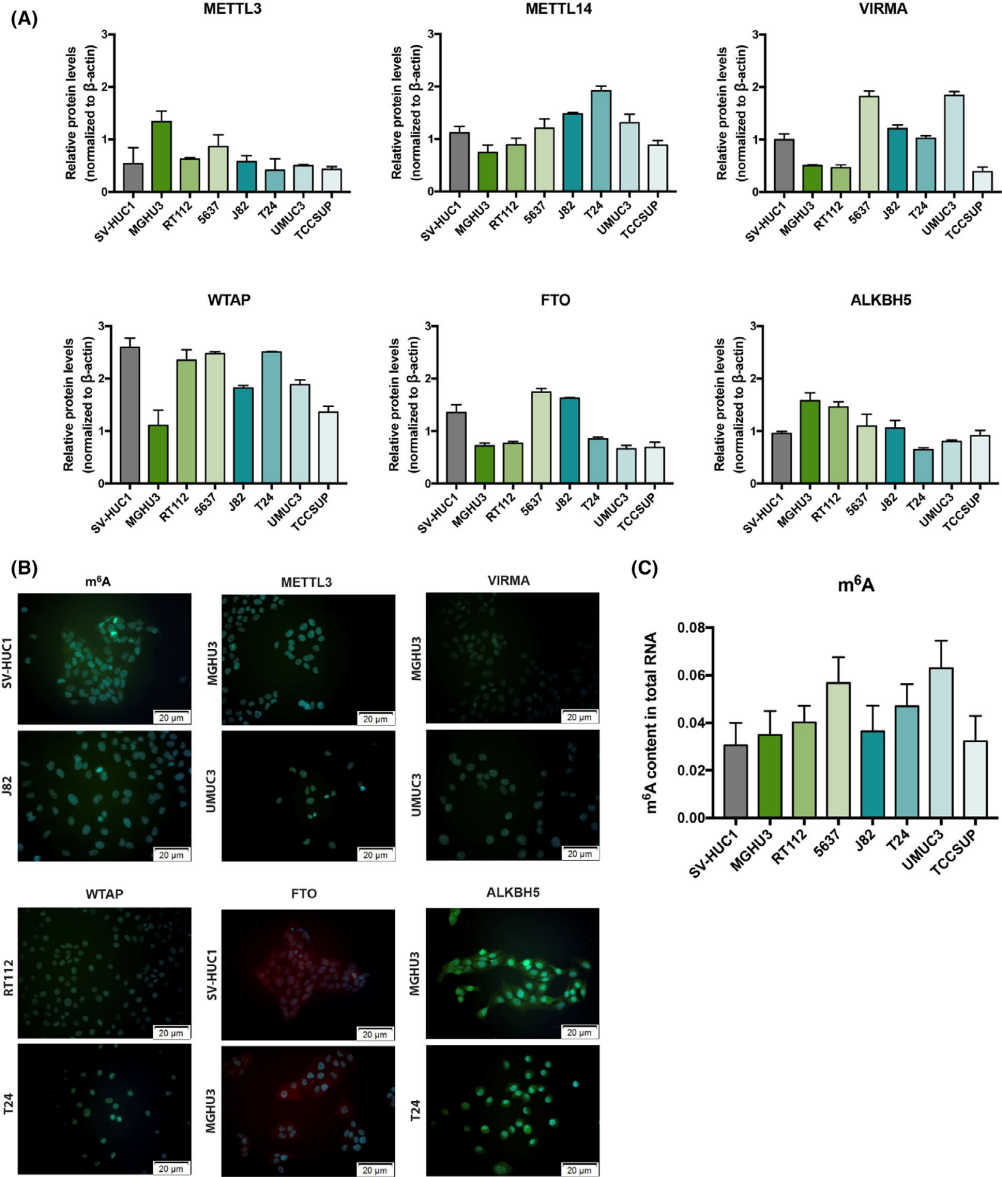
Characterization of bladder cancer cell lines: regulatory proteins (A) METTL3, METTL14, WTAP, FTO, and ALKBH5 protein levels in the same cell lines. SV‐HUC1 cell line was used as control. Experiments were performed in triplicates. Beta‐actin is presented as normalizer. (B) Illustrative images of immunofluorescence for all tested m^6^A regulatory proteins. Signal intensity was compared to the negative control. All pictures were obtained at 200× magnification, 50 µm. (C) Percentage of m^6^A in mRNA, using the ELISA m^6^A quantification kit. Data are shown as means ± SD and are representative of at least three independent experiments. Kruskal–Wallis test.

m^6^A regulatory proteins' expression significantly differed among the tested cell lines (Fig. 3A). Specifically, BlCa cell lines disclosed heterogenous levels of METTL14 writer comparing with benign cell line. Among BlCa cell lines, UMUC3 showed high METTL14 relative expression.

Overall, all writers and erasers were localized in the nucleus and cytoplasm, respectively, in all cell lines (Fig. 3B), which is in accordance with the observations in primary tumors using IHC. Illustrative examples of staining are depicted in Fig. S2.

Globally, BlCa cells exhibited higher m^6^A levels comparing with normal cells, although no statistically significant difference was found. The highest levels were observed in 5637 and UMUC3 cells, with approximately twice as much as the benign cell line, respectively, 0.55 and 0.6 vs. 0.3 approximately (Fig. 3C).

### 3.4 METTL14 knockdown in UMUC3: *in vitro* assays

Although METTL3 is the most studied catalytic subunit, METTL14, as one of the writers of m^6^A methyltransferase, is also actively involved in tumorigenesis.

**Fig. 4:**
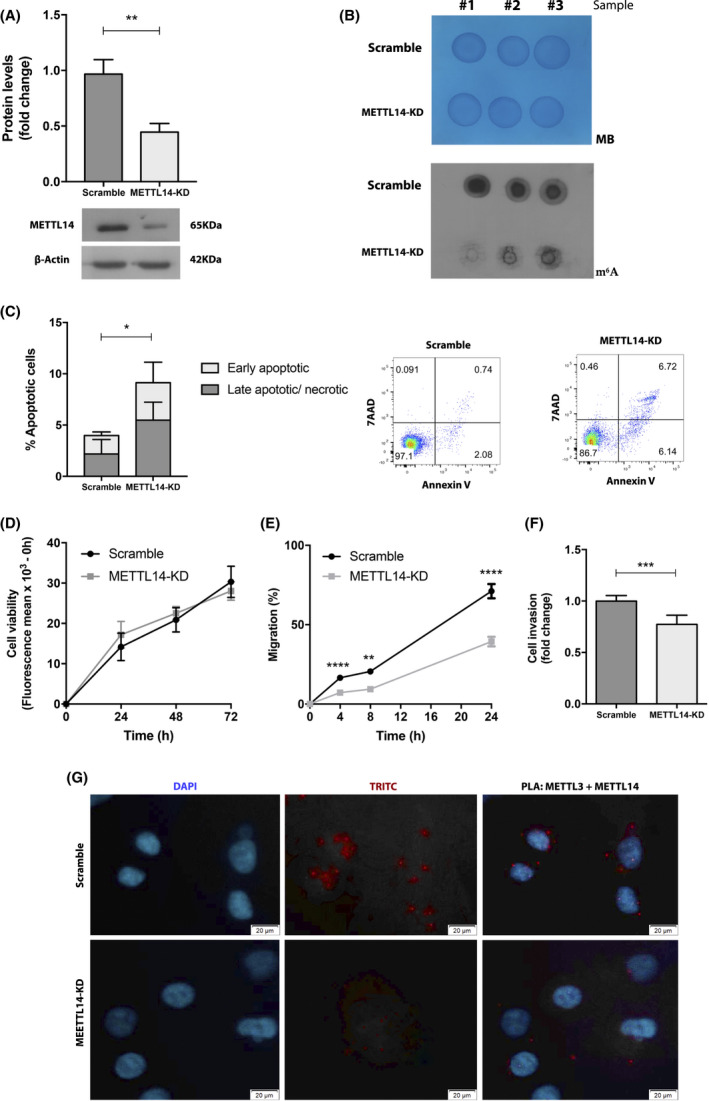
METTL14 Knockdown of UMUC3 cells (A) Efficiency in the protein expression of 55% in the condition of 1.5 µL Lipofectamine 300, Kruskal–Wallis test, *P* = 0.0041. (B) Reduction in m^6^A levels compared to the scramble condition. For loading control, 0.02% methylene blue. Phenotypic impact METTL14‐KD in UMUC3 cell line: (C) in apoptosis levels after 24 h (mean ± SD, *n* = 6) (D) in cell viability in three time points (E) in cell migration after 24 h (F) in cell invasion after 24 h. Data are shown as means ± SD and are representative of at least three independent experiments. Mann–Whitney *U*‐test: *P < 0.05, **P < 0.01, ***P < 0.001 and ****P < 0.0001. (G) Proximity ligation assay for UMUC3 and METTL14‐KD UMUC3 cells (400× magnification, 20 µm).

Because UMUC3 cell line showed a high relative expression s of METTL14, and it represents invasive urothelial carcinoma, an aggressive form of the disease, we decided to proceed with knockdown of this player in this specific cell line. METTL14‐KD was efficiently accomplished in UMUC3 by CRISPR‐Cas9 system (*P* = 0.0041) with a reduction of approximately 55% at the protein level (Fig. 4A), which was paralleled by decreased total m^6^A levels (Fig. 4B).

METTL14‐KD UMUC3 cells displayed significantly increased apoptosis at 24 h (about 3‐fold, *P* = 0.0152) (Fig. 4C), whereas no significant differences were observed in cell viability at the two different tested time points (Fig. 4D). Additionally, METTL14‐KD UMUC3 cells showed significantly reduced migration (24 h, *P* < 0.001) (Fig. 4E) and cell invasion capacity (24 h, *P* = 0.0006) (Fig. 4F).

Remarkably, the interaction between METTL14 and METTL3 was significantly reduced in METTL14‐KD UMUC3 cells compared to scramble (Fig. 4G).

### 3.5 METTL14 knockdown in UMUC3: *in vivo* assay

**Fig. 5:**
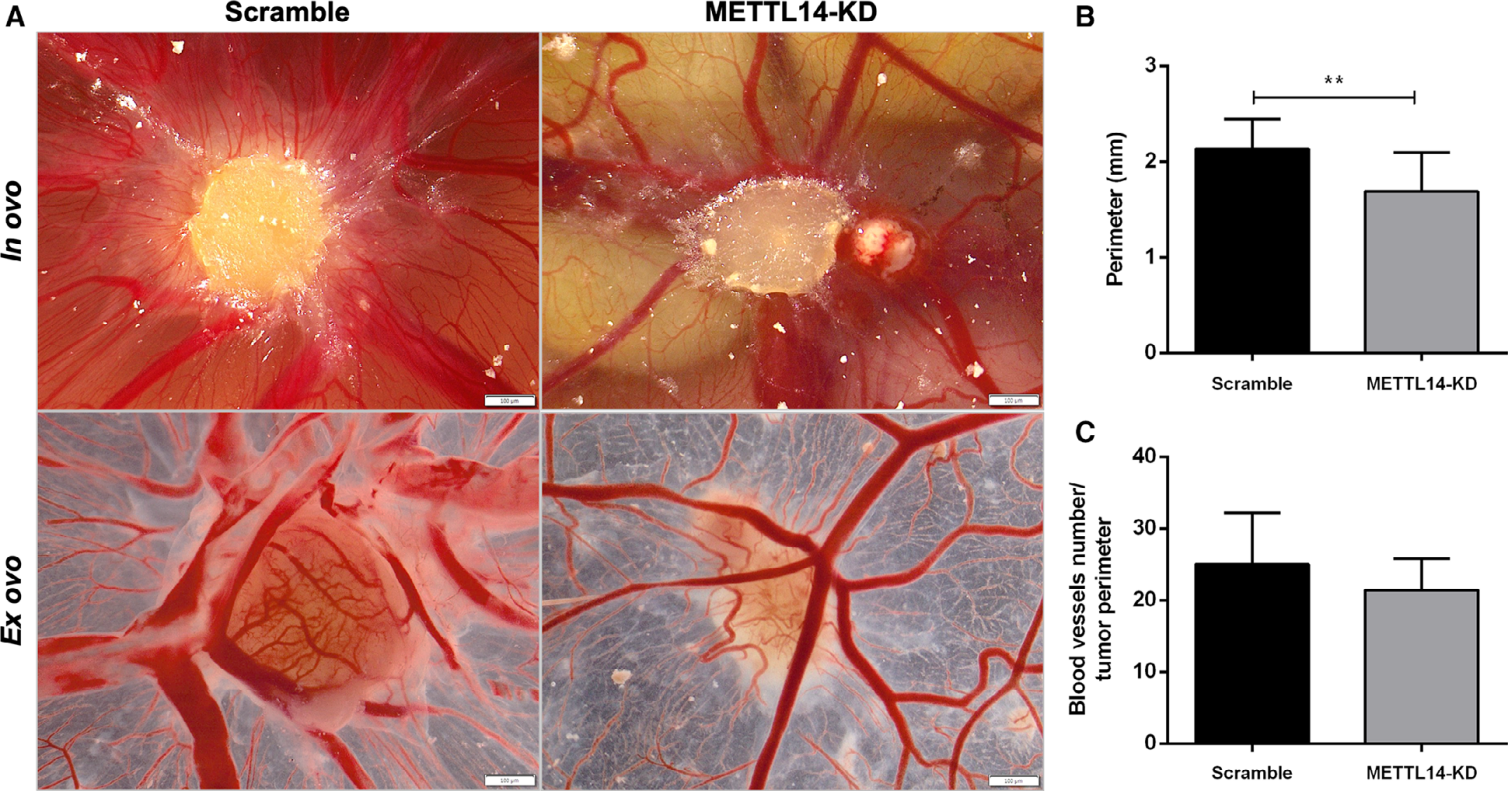
Knockdown of METTL14 in UMUC3 *in vivo* (A) Macroscopic view of tumor formation (*in ovo* and *ex ovo*) and neo‐angiogenesis in UMUC3 scramble and METTL14‐KD experimental conditions. Notice reduced size and decreased number of vessels in METTL14‐KD tumors. All pictures were obtained at 12.5× magnification, 100 µm. (B, C) Distribution of macroscopic tumor size and number of peri‐tumor vessels (normalized to size of tumor) in scramble and METTL14‐KD, reported as mean ± SD. *n* = 11 scramble condition; *n* = 11 METTL14‐KD tumors. Mann–Whitney *U*‐test: **P < 0.01.

To further understand the role played by METTL14 in BlCa tumor growth, we performed the CAM assay. METTL14‐KD cells generated significantly smaller tumors compared to scramble condition (Fig. 5A,B). Additionally, METTL14‐KD tumors disclosed a lower number of vessels at tumor periphery, even when normalizing for tumor size (Fig. 5C).

## 4 Discussion

Bladder cancer is one of the most incident urological neoplasms and its treatment remains a major health concern owing to the high cost of follow‐up and poor survival of patients with advanced‐stage disease. Hence, further insight into the biological mechanisms underlying disease progression is mandatory. m^6^A RNA methylation was firstly reported in 1974 by Ronald Desrosiers, but only recently its function began to be understood [36]. Overall, this modification affects numerous aspects of RNA metabolism, structure, function, and stability, playing a critical role in human diseases, including cancer [17]. Although m^6^A dysregulation has been reported in several tumors, data regarding BlCa are rather limited. Thus, we assessed m^6^A RNA abundance and expression of the respective regulatory proteins, aiming to correlate those findings with BlCa behavior.

The selection of the more informative m^6^A‐regulator proteins was performed by *in silico* analysis of RNA‐seq data from MIBC patients available at the TCGA database. Thus, METTL3, METTL14, VIRMA, WTAP (components of the m^6^A writer complex), and ALKBH5, FTO (components of the m^6^A eraser complex) surfaced as the mostly altered molecules of those involved in m^6^A regulatory network in BlCa. Nevertheless, from the six m^6^A‐regulator proteins, only METTL14 was found to be differentially expressed between tumors and adjacent normal samples, in TCGA dataset. In fact, Chen et al. [37] using mRNA expression data from The Cancer Genome Atlas (TCGA) m^6^A‐regulator proteins can contribute to bladder cancer progression.

Furthermore, protein expression of all the selected ‘writers’ and ‘erasers’ was evaluated by IHC in an independent set of BlCa and normal urothelium tissues from IPO Porto. In this series, instead of the ‘morphologically normal adjacent tissue’, normal urothelial tissue (NUT) from patients without UC was used, which constitutes an advantage. Indeed, owing to the widely acknowledged ‘field effect’ phenomenon occurring in urothelial carcinogenesis, ‘morphologically normal adjacent tissue’ might already harbor epigenetic and/or epitranscriptomic alterations. Remarkably, when comparing BlCa with NUT, we found that not only METTL14 but also METTL3, VIRMA, and ALKBH5 expression was significantly decreased in cancer tissues. Contrarily, m^6^A modification did not disclose significant differences between tumors and normal tissues, which might be explained by the different functions of m6A‐regulator proteins, independently of m6A modification [38, 39].

Interestingly, a significant association between reduced METTL3 expression and advanced pathological stage was found, which is in accordance with the correlation between lower METTL3 expression and higher tumor grade observed by others [37].

Emerging evidence suggests that m^6^A‐regulatory proteins can function either as oncogenes or tumor suppressors, depending on the tumor model and the specific cellular context. Indeed, METTL3, the most well‐described m^6^A writer associated with carcinogenesis, was found upregulated in leukemia, as well as pancreatic, breast, and lung cancers, whereas in endometrial carcinoma, glioblastoma, and prostate cancer it was reported as downregulated [17]. Concerning METTL14, a previous study in hepatocellular carcinoma suggested a tumor suppressor function [40], contrarily to the oncogenic role in pancreatic [41] and breast [42] cancers. Our findings suggest a key function of both METTL3 and METTL14 in BlCa, which is further supported by heterogeneity and significant reduction of the heterodimeric catalytic core METTL3/METTL14 in MIBC and NMIBC compared to NUT. Additionally, METTL3/METTL14 expression positively correlated with m^6^A RNA modification, as already described in different malignancies [24], emphasizing that writers' downregulation leads to disruption of the complex functionality and consequent reduction of global m^6^A levels. Indeed, in glioblastoma and hepatocellular carcinoma, METTL14 downregulation associated with m^6^A levels reduction [40, 43], while VIRMA upregulation positively correlated with higher m^6^A levels, in both testicular germ cell tumors and prostate cancer [21, 22].

Previously, METTL3 overexpression was reported in BlCa tissues, but this resulted from the comparison with ‘adjacent normal tissues’ [44, 45] and not true normal bladder urothelium, a feature that, as mentioned above, may strongly impact the results. Furthermore, the aforementioned studies used a rather limited number of cases comparatively to our study (*n* = 22 [44] and *n* = 56 [45], vs. *n* = 120).

Comparative analysis of tested cell lines showed higher m^6^A levels in BlCa cells compared to benign urothelial cell line. In the same panel of cells, METTL14 disclosed heterogeneous expression, with the highest levels observed in the more ‘aggressive’ cell lines. Importantly, METTL14‐KD led to significant m^6^A reduction and significantly inhibited migration and invasion traits of cancer cells. Thus, METTL14 seems to be required for the proper establishment of cellular m^6^A profile, being also a major promoter of BlCa aggressiveness. Furthermore, the oncogenic role proposed for METTL14 in BlCa was further supported by *in vivo* CAM assay, with significantly reduced tumor size and vessel density found in METTL14‐KD cells. We emphasize CAM assay's versatility for assessing *in vivo* tumor properties and microtumor aggressiveness features [46, 47, 48], also recently demonstrated by other researchers [49]. This is in line with reported findings in acute myeloid leukemia [50] although opposite findings were observed by others in BlCa [51]. Indeed, contrarily to our findings, Gu et al. observed decreased m^6^A content in BlCa. Nonetheless, the same authors showed increased METTL14 expression in patients with advanced stage of this malignancy, associating METTL14 overexpression with tumor aggressiveness. Surprisingly, the same study indicated that METTL14 knockout led to larger tumors and increased invasion. Indeed, different and sometimes contradictory results were also reported for kidney cancer concerning FTO [52, 53] and for METTL3 in prostate cancer [54, 55]. Therefore, clarifying epitranscriptomic target genes and related pathways is mandatory in future investigations to understand the mechanistic impact of m^6^A RNA methylation in carcinogenesis and tumor aggressiveness.

Regarding METTL14 enzymatic action, previous studies have shown that although METTL14 does not have a catalytic function *per se*, it forms a heterodimer with METTL3, which is required for the complex stabilization and activity [12, 15, 18]. Specific interactions between METTL14 methyltransferase domain‐MTD14 and METTL3 domain‐MTD3 are required for METTL3 catalytic activity. Hence, METTL3‐METTL14 forms a stable methyl capable heterodimer. Wang et al. advocated that not only METTL14 structurally supports the METTL3 catalytic cavity but it also plays a critical role in substrate RNA recognition [56]. This is in line with our findings in UMUC3 cells, in which METTL14‐KD was accompanied by a reduction in m^6^A levels, again suggesting METTL14 as a necessary partner for proper m^6^A establishment. Yet, considering the plethora of m^6^A ‘writers’ and ‘erasers’, the effects of altered expression of some factors might be compensated by variation of others. Herein, we found a significant positive correlation between ‘writers’ and ‘erasers’ expression, which might be a mechanism for feedback‐compensation of reduced m^6^A modification, also previously reported for hepatocellular carcinoma [40]. Considering the remainder factors, VIRMA transcript levels were reported to be significantly higher in hepatocellular carcinoma, [57], whereas in glioblastoma, ALKBH5 findings were in line with our observations in BlCa [43].

In summary, m^6^A‐regulator proteins are frequently dysregulated in BlCa. Notwithstanding the limitations of this study (e.g., its retrospective nature and subjectivity of IHC scoring), METTL3/METTL14 methyltransferase complex downregulation seems to dictate reduced m^6^A levels and contribute to cancer progression. Indeed, METTL14‐KD in UMUC3 cells resulted in significantly decreased invasiveness and increased apoptosis. Future studies should explore the usefulness of this player for the development of new therapeutic strategies.

## 5 Conclusions

Overall, m^6^A‐regulator proteins are frequently dysregulated in BlCa. Importantly, this is a comprehensive study on METTL14 function in BlCa, with evaluation of its expression in 120 human patient samples and in eight distinct cell lines, in addition to assessment of phenotypic effects of its knockdown on both viability, apoptosis, migration, and invasion properties. METTL14‐KD and consequent m^6^A downregulation reduced BlCa cells' malignant phenotype, suggesting an oncogenic role for METTL14 in BlCa. In the future, we intend to explore in detail the specific pathways and targets that are regulated by m^6^A deposit and/or removal, namely those related to EMT and cell cycle regulation. Recent work from our team and others has demonstrated the prognostic role of vimentin and E‐, P‐, and N‐cadherin in the EMT process in BlCa, so indicating that these and other players merit further investigation. Additionally, and owing to the observed increased apoptosis, we intend to evaluate the influence of METTL14 (and other players) in apoptosis‐related proteins (caspases family) and in anti‐apoptotic factors such as NF‐ĸB [58, 59, 60, 61].

## Conflict of interest

The authors declare no conflict of interest.

## Author contributions

RH and CJ conceptualized the study. CG‐T, JL, VM‐G, DB‐S, CM‐L, SM‐R, JPS, MP‐C, and IC contributed to methodology, analysis, and investigation. CJ provided resources. CG‐T wrote the original draft. DB‐S, JL, RH, and CJ wrote, reviewed, and edited the manuscript. RH and CJ supervised the study. CJ contributed to project administration. All authors have read and agreed to the published version of the manuscript.

### Peer Review

The peer review history for this article is available at https://publons.com/publon/10.1002/1878‐0261.13181.

## Supporting information


**Fig. S1.** Illustrative images of immunostaining: (1) m^6^A nuclear immunostaining in bladder cancer; (2) WTAP nuclear immunostaining in bladder cancer; (3) FTO nuclear immunostaining in bladder cancer. Immunostaining based on h‐score (ranges from 0, +1, +2, +3, +4, +6, +9).

**Fig. S2.** Illustrative images of western blot.

**Table S1.** Primary antibodies used in IHC.
**Table S2.** Clinicopathologic characterization of bladder cell lines.
**Table S3.** Primary antibodies used in western blot and immunofluorescence.

## Data Availability

All data generated or analyzed during this study are included in this published article and its supplementary information files.
