# Supplementary figures and images for: Downregulation of m6A writer complex member METTL14 in bladder urothelial carcinoma suppresses tumor aggressiveness
Source: Mol Oncol. 2022 Mar 24;16(9):1841–56. doi: 10.1002/1878-0261.13181 (PMC9067151; doi:10.1002/1878-0261.13181)

**A**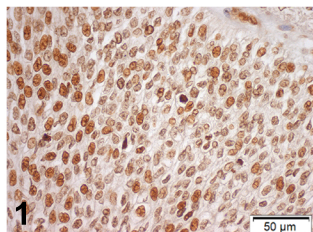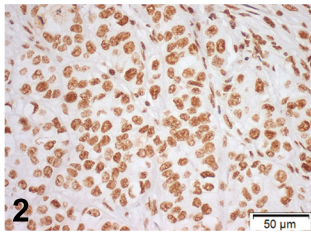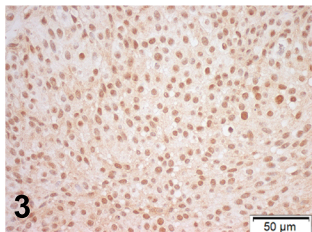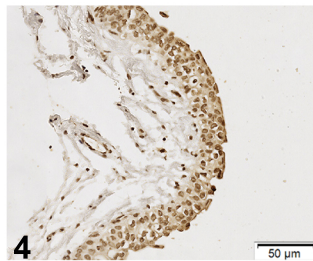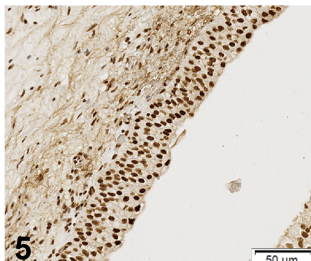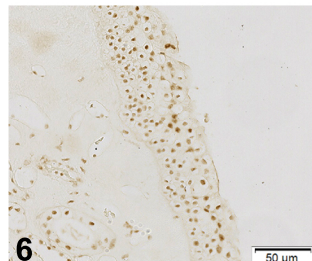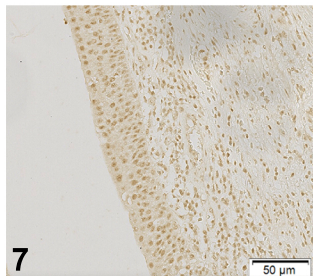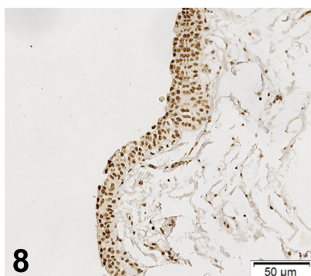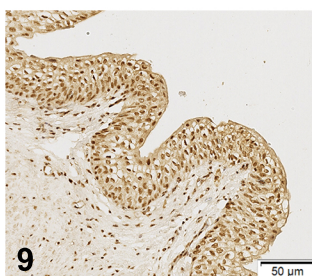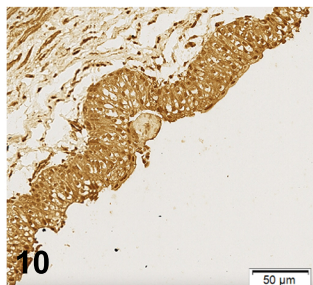

Supplement: Supplementary file 1 — Fig. S1. Illustrative images of immunostaining: (1) m6A nuclear immunostaining in bladder cancer; (2) WTAP nuclear immunostaining in bladder cancer; (3) FTO nuclear immunostaining in bladder cancer. Immunostaining based on h‐score (ranges from 0, +1, +2, +3, +4, +6, +9). [file MOL2-16-1841-s003.pdf]

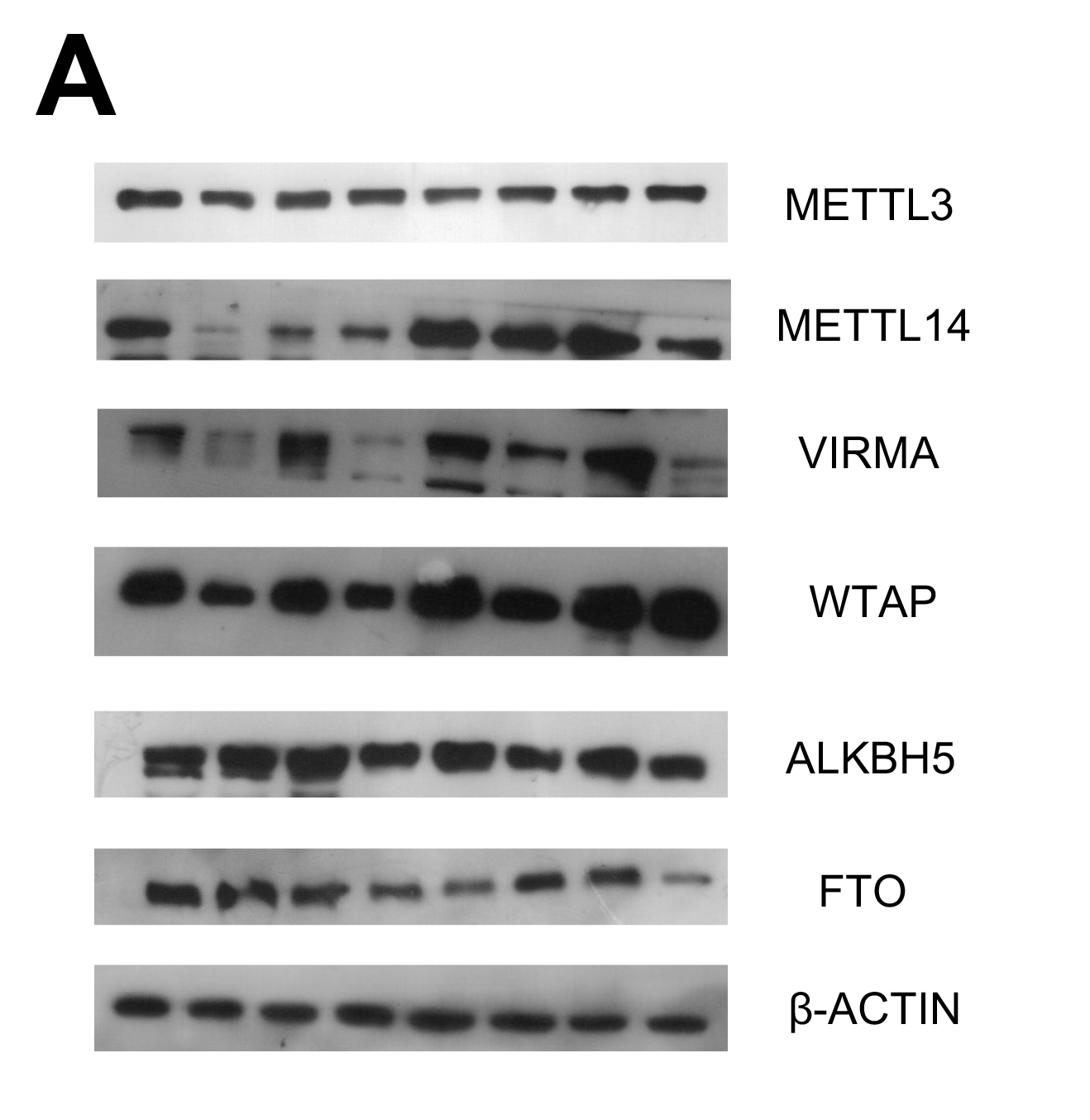

Supplement: Supplementary file 2 — Fig. S2. Illustrative images of western blot. [file MOL2-16-1841-s001.tiff]
