# Supplementary material for: Downregulation of m6A writer complex member METTL14 in bladder urothelial carcinoma suppresses tumor aggressiveness
Source: Mol Oncol. 2022 Mar 24;16(9):1841–56. doi: 10.1002/1878-0261.13181 (PMC9067151; doi:10.1002/1878-0261.13181)
Supplement: Supplementary file 3 — Table S1. Primary antibodies used in IHC. Table S2. Clinicopathologic characterization of bladder cell lines. Table S3. Primary antibodies used in western blot and immunofluorescence. [file MOL2-16-1841-s002.pdf]

Guimarães-Teixeira et al._supplementary data

Supplementary Tables

Table S1. Primary antibodies used in IHC

Antibodies	Vendor	Catalog number	Positive control	Antigen retrieval	Primary antibody dilution
**Anti- METTL3 [EPR18810]**	Abcam, Cambrige, United Kingdom	ab195352	Normal testicle	EDTA buffer microwave (1mM, pH=8)	1:500 overnight 4ºC
**Anti- METTL14 [CL4252]**	Abcam, Cambrige, United Kingdom	ab220031	Normal testicle	Citrate buffer microwave (10mM, pH=6)	1:750 overnight 4ºC
**Anti- WTAP [EPR18744]**	Abcam, Cambrige, United Kingdom	ab195380	Normal testicle	EDTA buffer microwave (1mM, pH=8)	1:500 overnight 4ºC
**Anti-VIRMA/ Virilizer**	Cell Signaling Technology, United States, USA	88358	Normal testicle	Citrate buffer microwave (10mM, pH=6)	1:200 overnight 4ºC
**Anti-FTO [5-2H10]**	Abcam, Cambrige, United Kingdom	ab92821	Normal testicle	Citrate buffer microwave (10mM, pH=6)	1:500 overnight 4ºC
**Anti- ALKBH5**	Proteintech Europe, United Kingdom	16837-1-AP	Normal testicle	Citrate buffer microwave (10mM, pH=6)	1:500 overnight 4ºC
**Anti-N6-methyladenosine (m^6A)**	Abcam, Cambrige, United Kingdom	ab190886	Normal brain	EDTA buffer water bath (1mM, pH=8)	1:750 overnight 4ºC

Table S2. Clinicopathologic characterization of bladder cell lines

Cell Line	Type	Grade[a]	Stage[a]	Subtype[a]	Gender	Media
**SV-HUC1**	normal	n/a	n.a.	n.a.	male	F12-K
**MGHU3**	tumor	G1	pTa/T1	luminal	male	DMEM
**RT112**	tumor	G2	pTa	luminal	female	RPMI
**5637**	tumor	G2	pTx	mixed	male	RPMI
**J82**	tumor	G3	pT3	basal	male	MEM
**T24**	tumor	G3	pTa	basal	female	RPMI
**UMUC3**	tumor	G3	pT2- T4	basal	male	DMEM
**TCCSUP**	tumor	G4	pTx	basal	female	MEM

n.a.- not applicable
a- according to **(1)**

Table S3. Primary antibodies used in Western blot and Immunofluorescence

Antibodies	Vendor	Catalog number	Western-blot dilution	Primary antibody: incubation time	Secondary antibody specie	Blocked	IF dilution
**Anti-METTL3**	Abcam, Cambrige, United Kingdom	ab195352	1:1000	1h at RT	Anti-rabbit	BSA 5%	1:250
**Anti-METTL14**	Abcam, Cambrige, United Kingdom	ab220031	1:1000	1h at RT	Anti-mouse	BSA 5%	n.a.
**Anti-WTAP**	Abcam, Cambrige, United Kingdom	ab195380	1:1000	1h at RT	Anti-rabbit	BSA 5%	1:100
**Anti-VIRMA / Virilizer**	Cell Signaling Technology, United States, USA	88358	1:1000	1h at RT	Anti-rabbit	BSA 5%	1:100
**Anti-FTO**	Abcam, Cambrige, United Kingdom	ab92821	1:1000	1h at RT	Anti-mouse	Milk 5%	1:100
**Anti-ALKBH5**	Proteintech Europe, United Kingdom	16837-1-AP	1:1000	1h at RT	Anti-rabbit	Milk 5%	1:500
**β-Actin**	Sigma-Aldrich	A1978	1:10 000	1h at RT	Anti-mouse	BSA 5%	n.a.

n.a. – not applicable; RT – room temperature
